# Ginkgo Biloba Ameliorates Subfertility Induced by Testicular Ischemia/Reperfusion Injury in Adult Wistar Rats: A Possible New Mitochondrial Mechanism

**DOI:** 10.1155/2016/6959274

**Published:** 2016-12-22

**Authors:** Asmaa Ibrahim Ahmed, Noha N. Lasheen, Khaled Mohamed El-Zawahry

**Affiliations:** ^1^Anatomy, Faculty of Medicine, Ain Shams University, Cairo, Egypt; ^2^Physiology, Faculty of Medicine, Ain Shams University, Cairo, Egypt; ^3^Dermatology and Andrology, Faculty of Medicine, Ain Shams University, Cairo, Egypt

## Abstract

Testicular torsion, a surgical emergency, could affect the endocrine and exocrine testicular functions. This study demonstrates histopathological and physiological effects of testicular ischemia/perfusion (I/R) injury and the possible protective effects of Ginkgo biloba treatment. Fifty adult male Wistar rats, 180–200 gm, were randomly divided into sham-operated, Gingko biloba supplemented, ischemia only, I/R, and Gingko biloba treated I/R groups. Overnight fasted rats were anaesthetized by Pentobarbital; I/R was performed by left testis 720° rotation in I/R and treated I/R groups. Orchiectomy was performed for histopathological studies and detection of mitochondrial NAD^+^. Determination of free testosterone, FSH, TNF-*α*, and IL1-*β* in plasma was performed. Plasma-free testosterone was significantly decreased, while plasma FSH, TNF-*α*, IL-1*β*, and testicular mitochondrial NAD^+^ were significantly increased in I/R group compared to control group. These parameters were reversed in Gingko biloba treated I/R group compared to I/R group. I/R caused marked testicular damage and increased APAF-1 in the apoptotic cells which were reversed by Ginkgo biloba treatment. It could be concluded that I/R caused subfertility induced by apoptosis and oxidative stress manifested by the elevated testicular mitochondrial NAD^+^, which is considered a new possible mechanism. Also, testicular injury could be reduced by Gingko biloba administration alone.

## 1. Introduction

Testicular torsion, an abnormal twisting of spermatic cord or testicular rotation, represents a surgical emergency affecting mainly adolescents, probably following trauma to the scrotum, or may occur after strenuous exercise [1]. It could be manifested by acute severe scrotal pain and low grade fever. The twisted spermatic cord might cause a decrease or complete loss of blood flow to the affected testis [2]. It is valuable to determine the degree of twisting as it could result in progressive damage in both testicular structure and functions. Hence a rapid surgical maneuver should be performed to counterrotate both testes and spermatic cord to allow reperfusion [3]. This acute scrotal pain could occur intermittently followed by spontaneous resolution [4]; therefore, these cases should be surgically evaluated to prevent further torsion [5]. On the other hand, ipsilateral testicular torsion could affect the contralateral testis [4].

Koksal et al. [6] reported that the testicular injury following torsion has both ischemic and reperfusion components. The reperfusion injury could be more severe than ischemic injury [7]. The reperfusion injury resulting from toxic-free radicals, such as superoxide anions, hydroxyl radicals, and nitric oxide (NO), could damage the DNA structure [8] and could be a major factor in the etiology of male infertility [9].

Moreover, apoptosis, a physiologic process that entails the programmed cell death indicating tissue injury, is essential during normal spermatogenesis [10]. However, testicular I/R injury usually caused an extraordinary germ cells apoptosis [8], vacuolization of the seminiferous epithelium, and decreased sperm production [11].

Li et al. [12] mentioned that EGb 761 was a standardized extract of Ginkgo biloba leaves. It became commonly used for the treatment of cardiovascular disorders, diabetes mellitus, and aging [13]. The effects of Ginkgo biloba on ischemia/reperfusion (I/R) were studied on liver, brain, peripheral nerves, testes, intestine, and lung [14].

Later, Kanter [15] reported that Ginkgo biloba administration could be adjunctive therapy to surgical repair for rescuing the testis from I/R injury, thereby being a clinically important goal. The antioxidant effects of Ginkgo biloba could be due to its bioflavonoids contents, which might protect the tissues against free radicals-induced tissue damage [16]. It could, also, prevent cell damage resulting from ischemia and hypoxia, possibly by inhibiting the platelet activating factor; thus, it could be effective on cardiovascular, respiratory, and central nervous system and renal systems [17]. In addition, Singh et al. [13] reported that the organic acids of Ginkgo biloba extract were responsible for its antioxidant, antiallergic, anti-inflammatory, antiproliferative, antianxiety, and anticarcinogenic effects.

All previous studies in rat model of unilateral testes torsion/detorsion focused on the Ginkgo biloba extract effect on the antioxidant enzyme levels. The present work comprehensively investigated the morphometric parameters, mitochondrial function (NAD^+^), and apoptotic changes of I/R on the testis and the possible protective effects of Ginkgo biloba extract regarding testicular structure and exocrine and endocrine functions after unilateral testicular torsion/detorsion.

*Aim of the Work*. This study was designed to investigate histopathological changes in addition to exocrine and endocrine testicular functions caused by torsion/detorsion induced by experimental testicular ischemia/reperfusion injury. The possible effects of Ginkgo biloba were studied, in addition to the possible underlying mechanism(s).

## 2. Materials and Methods

*Drugs*. Ginkgo biloba was obtained from El Amriya Pharmaceutical Industries (Alexandria, Egypt); its concentration was 40 mg/ml.

### 2.1. Experimental Protocol

#### 2.1.1. Animals

Fifty adult male Wistar rats, weighing 180–200 grams, were purchased from the Animal Farm, Helwan, Cairo, Egypt, and housed in animal cages (5 rats/cage) with suitable ventilation, temperature of 22–25°C, 12 hours light dark cycle, and free access to food and water, ad libitum, in the Animal House, Medical Research Unit, Faculty of Medicine, Ain Shams University. Rats were allowed to adapt to the new environment for 7 days prior to experimental procedures to decrease the possible discomfort. Unnecessary pain or stress was avoided and animal manipulation was performed with maximal care and hygiene. All rats received human care according to the criteria outlined in the “Guide for the Care and Use of Laboratory Animals” prepared according to guidelines of animal use of the Ethical committee of Ain Shams University.

Rats were divided randomly into the following groups:

*Group I: main control group (n* = 20) which were further subdivided into
*Group Ia: sham-operated group (negative control) (n* = 10*):* they were exposed to the procedure of ischemia/reperfusion without induction of torsion/detorsion (the left testicular artery and vein were not occluded by clamping); then orchiectomy was performed.
*Group Ib: Ginkgo biloba supplemented group (positive control) (n* = 10): they received Gingko biloba extract 50 mg/kg by gavage as a single dose [15]. They were exposed to the procedure of ischemia/reperfusion without induction of torsion/detorsion (the left testicular artery and vein were not occluded by clamping); then orchiectomy was performed.
*Group II: ischemia only group (n* = 10): they were exposed to torsion for 2 hours only [7]; then, orchiectomy was performed.
*Group III: ischemia/reperfusion group (n* = 10): they were exposed to testicular ischemia (torsion) for 2 hours followed by reperfusion (detorsion) for 2 hours [18]; then orchiectomy was performed.
*Group-IV: Ginkgo biloba treated ischemia/reperfusion group (n* = 10): rats in this group were exposed to torsion for 2 hours, followed by detorsion for 2 hours. They received Gingko biloba extract in a dose of 50 mg/kg by gavage 40 minutes before detorsion [15]. At the end of I/R period, orchiectomy was performed. The Gingko biloba dose was equivalent to maximum therapeutic human dose and was calculated according to Paget and Barnes [19].

On the day of sacrifice, overnight fasted rats were anaesthetized with intraperitoneal injection of Pentobarbital (40 mg/kg BW).

### 2.2. Testicular Ischemia/Reperfusion Procedure

The scrotum was entered through a paramidline incision. The tunica vaginalis was opened, and the left testis was delivered to the surgical field. The left testis was rotated 720° in a clockwise direction [7]. Then, the left testicular artery and vein were occluded with a microvascular clamp to induce ischemia for 2 hours followed by clamp removal to induce reperfusion for another 2 hours [18]. Then, after, orchiectomy was performed.

An abdominal midline incision was performed. The collected blood samples from the abdominal aorta into heparinized tubes were centrifuged at 4000 rpm for 15 min., to separate plasma for subsequent determination of free testosterone, FSH, TNF-*α*, and IL1-*β*.

The left testes from all groups were dissected and divided into two parts: one used for determination of mitochondrial NAD^+^ as one of markers of oxidative stress, and the other part was subjected to histological examination.

### 2.3. Evaluation of Mitochondrial NAD^+^ in Testicular Tissue

Mitochondria isolation was from testicular tissues which were stored frozen at −80°C till the day of NAD^+^ determination, according to N. K. Saleh and H. A. Saleh [20]. Hydrolysis of mitochondrial nicotinamide adenine dinucleotide (NAD^+^) directly reflects mitochondrial pores opening. The NAD^+^ was measured after perchloric acid extraction. in the case of isolated mitochondria, 0.1 ml of 21% (v/v) perchloric acid was added to 1 mg of protein/ml suspensions. The concentration of NAD^+^ in the perchloric acid extract of the testicular mitochondria was measured using an alcohol dehydrogenase reaction. The reaction mixture contained 1000 *μ*L of buffer-substance (0.1 M Tris acetate [pH 8.8] and 0.5 M ethanol), 100 *μ*L of the tissue extract neutralized, and 20 *μ*L of alcohol dehydrogenase. The reaction was initiated by enzyme addition and change of absorbance at 340 nm was recorded by a spectrophotometer.

*Determination of Free Testosterone Level in Plasma*. Determination of free testosterone level in plasma was done by using ELISA kits supplied by ABCAM, Egypt, while plasma FSH level was determined by IRMA technique according to Santner et al. [21]. 

*Determination of Plasma TNF-α*. Plasma TNF-*α* was determined by using the RayBio® Rat TNF-*α* ELISA kit (RayBiotech, Inc., Norcross, Georgia, USA).

*Determination of Plasma IL1-β*. Plasma IL1-*β* was determined by using ELISA kit (MABTECH® Rat), Egypt.

### 2.4. Histological Examination of Testes

#### 2.4.1. Light Microscopic (L/M) Study

*(1) Hematoxylin and Eosin (H&E) Stain*. Specimens from left testes of rats were immediately fixed into Bouin's fixative. Tissues were processed for preparation of paraffin blocks to get paraffin sections (5 *μ*m in thickness), which were stained by conventional Hematoxylin and Eosin (H&E) stain [22]. The testicular tissue was examined and evaluated in random order under blind conditions with standard light microscopy. Three slides prepared from the upper, lower, and mid-portions of the testes were evaluated completely for each testis.

*(2) Immunohistochemical Study (Apaf-1 Staining)*. The paraffin sections were used for detection of the Apoptosis protease-activating factor 1 (Apaf-1) expression. Immunostaining was performed with a Leica Bond-Max automatic immunostainer (Leica, Bannockburn, IL, USA) [23]. Apaf-1, an important part of apoptosome, is a protein playing a central role in apoptosis [24]. When apoptosis is stimulated, Apaf-1 binds with cytochrome C and procaspase 9, and then caspase-3 and caspase-7 become activated. This complex is called apoptosome resulting in the loss of the cell [25]. Cellular stresses, such as hypoxia, lead to caspase activation resulting in apoptotic cell death [24].

*(3) Preparation of Specimens for the Semithin Sections Toluidine Blue Stain*. Testes were immediately cut into cubes (1 mm in diameter) and fixed overnight in 2.5% phosphate-buffered glutaraldehyde (pH 7.3) at 4°C. Fixation in 1% buffered osmium tetroxide for 1-2 h was followed by dehydration in ascending grades of ethyl alcohol and clearance in propylene oxide and finally embedded in fresh Epon capsules. Semithin sections, 1 *μ*m in thickness, were cut with a glass knife and stained with toluidine blue and then examined by an Olympus light microscope [26]. 

*(4) Sperm Abnormality Test by Nigrosin and Eosin Stain*. The sperm abnormality was studied as described by Wyrobek et al. [27] as follows. The heads of the epididymis were excised and minced with small scissor and remained in 2 ml isotonic saline. Smears were prepared and stained with equal amount of 5% Nigrosin and 1% Eosin for 30 minutes. Three drops of the prepared smear were allowed to drop on the edge of the precleaned slide and by the edge of other clean slide; the smear was spread over the entire surface of the slide, which was left to dry and coded for subsequent microscopic examination. One thousand sperms were examined under oil immersion lens of light microscope for each rat and abnormal sperms were recorded and photographed. 

*(5) Computerized Morphometric Analysis (Image Analysis)*

*Quantitative Morphometric Measurements*. Sections were examined by using Leica DM2500 microscope with built-in camera (Weltzelar, Germany). All images were digitally acquired using an image analyser Leica Q win V.3 program (Weltzelar, Germany) installed on a computer in Histology Department, Faculty of Medicine, Ain Shams University. Five different nonoverlapping fields from five different stained sections of five different rats were examined in each group for measuring each of the following:

1. Mean seminiferous tubule diameter (MSTD) measured in micrometers.
2. Number of the primary spermatocytes per cross section of a seminiferous tubule.
3. Number of the rounded spermatids per cross section of a seminiferous tubule.
4. The rounded spermatids/primary spermatocytes ratio.
5. Number of the interstitial cells of Leydig cells per space.

All measurements were performed at high power field of magnification (×400); all data were collected, revised, and subjected for statistical analysis.

### 2.5. Statistical Analysis

All results in the present study were expressed as mean ± SE of the mean. Statistical Package for the Social Sciences (SPSS, Inc., Chicago, IL, USA) program, version 20.0, was used to compare significance between each of the two groups. One-way ANOVA for difference between means of different groups was performed on results obtained in the study. Differences were considered significant when *P* ≤ 0.05.

## 3. Ethics Committee

This study was approved by the Ethics Committee of Faculty of Medicine, Ain Shams University.

## 4. Results

### 4.1. Biochemical Results

**Figure 1:**
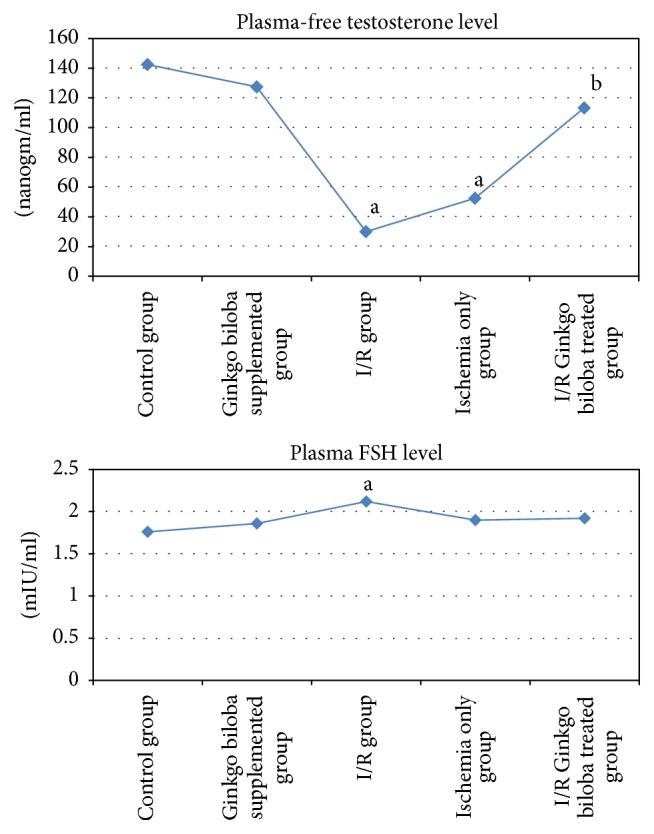
Plasma levels of free testosterone (nanogm/ml) and FSH (mIU/ml) in the different studied groups. (a) Significance by LSD at *P* < 0.05 from control group. (b) Significance by LSD at *P* < 0.05 from I/R group.

As shown in Table 1 and Figure 1, plasma-free testosterone was significantly decreased in ischemia/reperfusion (I/R) and ischemia only groups compared to negative control group (30 ± 11.55, 52.5 ± 10.31 versus 142.5 ± 29.83, *P* < 0.005, *P* < 0.01, resp.); however, it was nonsignificantly changed in both Ginkgo biloba treated I/R and Ginkgo biloba supplemented groups compared to negative control group. Compared to I/R group, Ginkgo biloba treated I/R group showed significant increase in plasma-free testosterone level (113.33 ± 34.8 versus 30 ± 11.55, *P* < 0.05), while it was insignificantly changed in ischemia only group.

Plasma FSH level was significantly elevated in I/R group compared to negative control group (2.12 ± 0.17 versus 1.76 ± 0.03, *P* < 0.02); however nonsignificant changes were observed in plasma FSH in ischemia only, Gingko biloba treated I/R, and Gingko biloba supplemented groups compared to the negative control group. Also, compared to I/R group, plasma FSH were insignificantly changed in ischemia only and Gingko biloba treated I/R groups, as shown in Table 1 and Figure 1.

**Figure 2:**
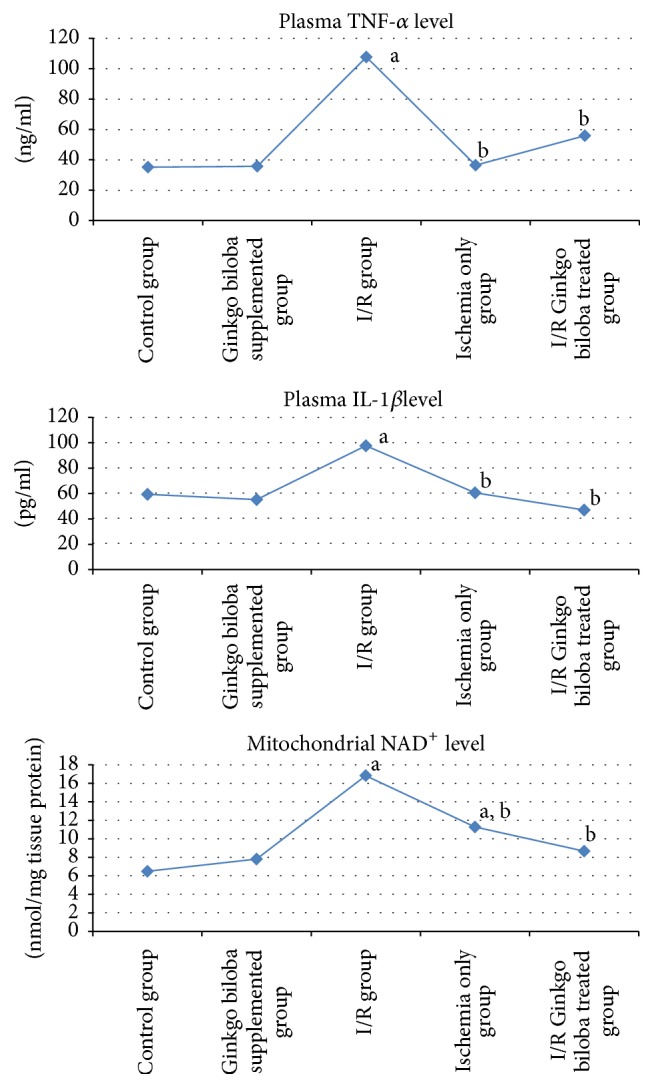
Plasma levels of mitochondrial NAD^+^ (nmol/mg tissue protein), TNF-*α* (ng/ml), and IL-1*β* (pg/ml) in the different studied groups. (a) Significance by LSD at *P* < 0.05 from control group. (b) Significance by LSD at *P* < 0.05 from I/R group.

Oxidative stress was studied by assessment of mitochondrial NAD^**+**^ in testicular tissue, which was significantly increased in ischemia only and I/R groups compared to control group (11.29 ± 0.92, 16.83 ± 1.53 versus 6.5 ± 1.09, *P* < 0.05, *P* < 0.001, resp.); however, insignificant changes were observed in mitochondrial NAD^+^ in Ginkgo biloba supplemented and Gingko biloba treated I/R groups compared to negative control group. In addition, it was significantly reduced in ischemia only group and Ginkgo biloba treated I/R group compared to I/R group (11.29 ± 0.92, 8.67 ± 1.58 versus 16.83 ± 1.53, *P* < 0.01, *P* < 0.001, resp.), as shown in Table 2 and Figure 2.

Regarding inflammatory and apoptotic factors, plasma TNF-*α* was significantly increased only in I/R group compared to the negative control group (107.65 ± 19.78 versus 35.15 ± 0.51, *P* < 0.001); however, insignificant changes were present in ischemia only, Gingko biloba treated I/R, and Gingko biloba supplemented groups compared to the negative control group. Compared to I/R group, plasma TNF-*α* was significantly reduced in ischemia only and Gingko biloba treated I/R groups (36.38 ± 1.4, 55.88 ± 6.22 versus 107.65 ± 19.78, *P* < 0.001 for each), as shown in Table 2 and Figure 2. Also, plasma IL-1*β* was significantly elevated in I/R group compared to the negative control group (97.71 ± 19.08 versus 59.39 ± 0.53, *P* < 0.01); however, it was insignificantly changed in ischemia only, Gingko biloba treated I/R, and Gingko biloba supplemented groups compared to the negative control group. Plasma IL-1*β* was significantly decreased in ischemia only and Gingko biloba treated I/R groups compared to I/R group (60.57 ± 1.17, 47.04 ± 0.07 versus 97.71 ± 19.08, *P* < 0.02, <0.002, resp.), as shown in Table 2 and Figure 2.

### 4.2. Histological Results

Histological examination of the testes of control rats (negative and Ginkgo biloba supplemented) groups (groups Ia and Ib) showed the same structure; therefore figures for the negative control group are representative for both.

**Figure 3:**
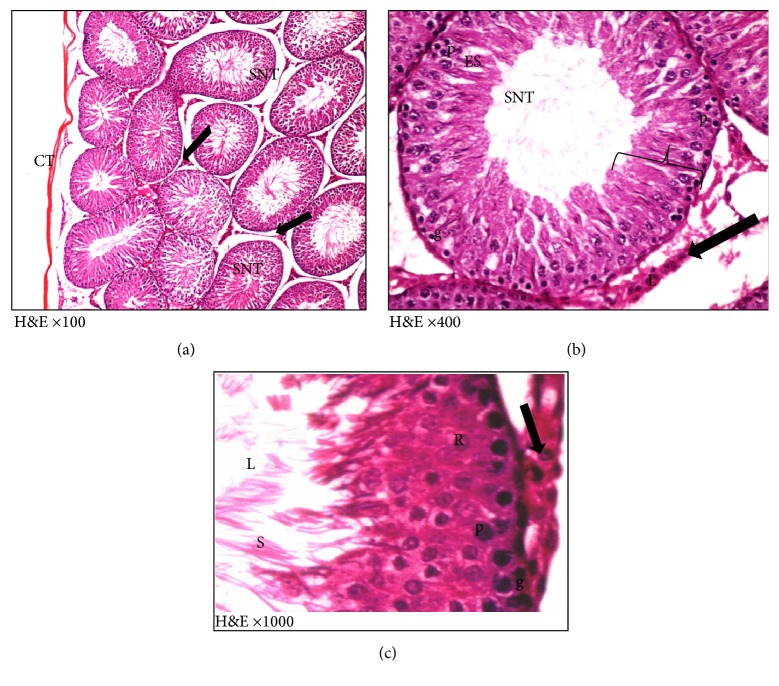
Photomicrographs of a cross section of adult control rat tests showing (a) normal connective tissue capsule (CT), normal architecture of the seminiferous tubules (SNT), and narrow interstitial space (↑) in between (×100). (b) The normal thickness of the germinal epithelium ( } ) of the seminiferous tubule (SNT). The presence of elongated spermatids (ES) lining the lumen and Leydig cells (L) in the interstitial tissue (↑) (×400). (c) The normal different layers of the germinal epithelium. g: spermatogonia, R: round spermatid, S: sperms, L: lumen, and P: primary spermatocytes; the (ES) elongated spermatids, Leydig cells in the interstitial tissue (↑) (×1000).

**Figure 4:**
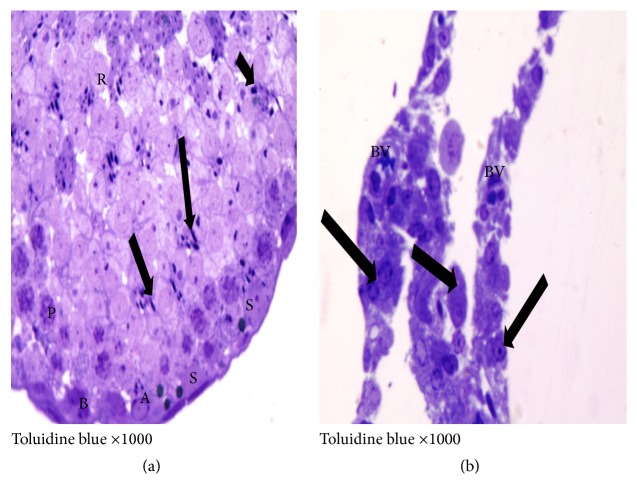
Photomicrographs of a semithin section of adult control rat tests (toluidine blue ×1000) showing (a) normal seminiferous tubule with its cellular components: A, spermatogonia; B, spermatogonia; P, primary spermatocytes; R, round spermatids; S, Sertoli cell adjoining the basement membrane. (b) The interstitial tissue containing Leydig cells (↑) arranged around blood vessels (BV).

H&E and toluidine blue stained testicular sections of the control group showed normal pattern of testicular structure, enclosed by fibrous connective tissue capsule and fibrous septa dividing the gland into lobules composed of multiple rounded seminiferous tubules with regular outlines, lined by 4–6 layers of germinal epithelium at different stages of spermatogenesis. The flagella of mature sperms, which had whorly appearance, were observed filling the lumens of the seminiferous tubules. The interstitial spaces of the tubules contained Leydig cells and some blood capillaries. The lining epithelium consists of germinal and Sertoli cells, which appeared pyramidal in shape and resting on the basement membrane. The germinal epithelium consists of spermatogonia resting on the basement membrane, large primary spermatocytes, early or rounded spermatids with acrosomal caps, and late or elongated spermatids (Figures 3 and 4).

**Figure 5:**
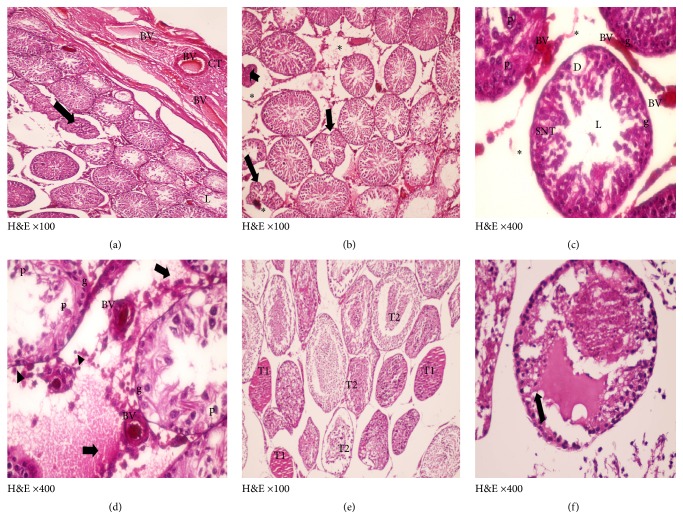
Photomicrographs of a cross section of ischemia only group adult rat tests (H&E) showing (a) mild congestion of the connective tissue capsule (CT) with dilated congested blood vessels (BV) with some shrunken seminiferous tubules (↑) and wide peritubular space (*∗*) and others with thin germinal epithelium and wide lumina (L) (×100). (b) Festoon appearance of tubules with detached basement membrane (↑), shrunken tubules (upwards black arrow) and wide peritubular space (*∗*) (×100). (c) Marked loss of general architecture of the seminiferous tubules (SNT) with loss of stratification of germ cells, area of depleted germ cells (D), scanty or hardly detected elongated mature spermatids, degenerated spermatogonia (g), and primary spermatocytes (P) with observed wide large interstitial tissue spaces (*∗*) and congested blood vessels (BV) (×400). (d) Severely affected tubules with degenerated spermatogonia (g) and primary spermatocytes (P), further lost stages of spermatogenesis, dilated congested blood vessels (BV) together with pyknotic nuclei of some Leydig cells (▲) embedded in acidophilic exudates (↑) (×400). (e) Some seminiferous tubules were filled with acidophilic exudates (T1) while other tubules showed exfoliated cells in their lumina (T2) (×100). (f) One of the tubules showing degenerated germ cells floating in the acidophilic exudates (↑) (×400).

**Figure 6:**
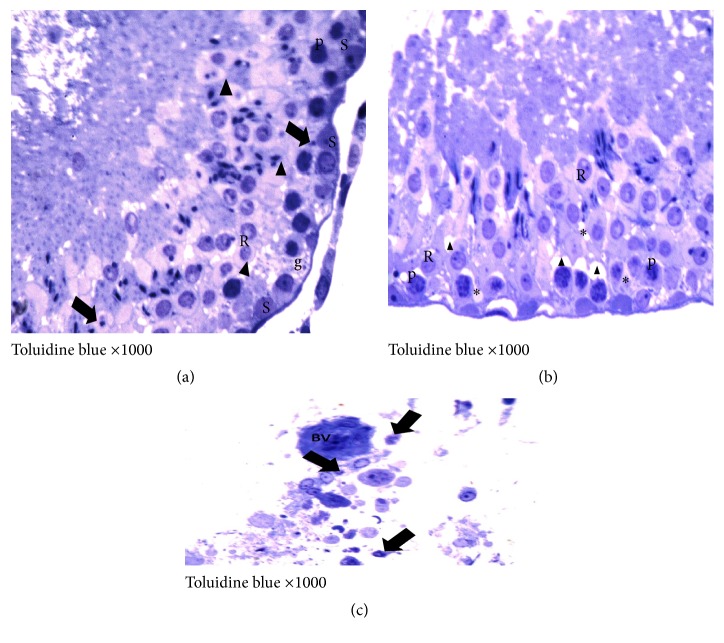
Photomicrographs of a semithin section of adult rat tests of ischemia only group showing (a) part of seminiferous tubule presenting degenerated spermatogonia (g) and pyknosis (↑), karyolysis (▲) of some primary spermatocytes (P), round spermatids (R), and apparently normal Sertoli cells nuclei (S) (×1000). (b) Intercellular (**∗** **)** and intracellular vacuoles (▲) in the primary spermatocytes (P) and round spermatids (R) (×1000). (c) Ill-defined cell membrane and degenerated cytoplasm are observed together with pyknotic nuclei of some Leydig cells (↑) arranged around blood vessel (BV) (×1000).

In ischemia only group, mild to moderate degeneration of the germinal epithelial lining of the seminiferous tubules was observed, in addition to interstitial edema, congestion of the interstitial blood vessels, and separation of the germinal epithelium with pyknotic nuclei of some Leydig cells that are embedded in acidophilic vacuolated exudates, some seminiferous tubules filled with an acidophilic exudates (Figures 5 and 6).

**Figure 7:**
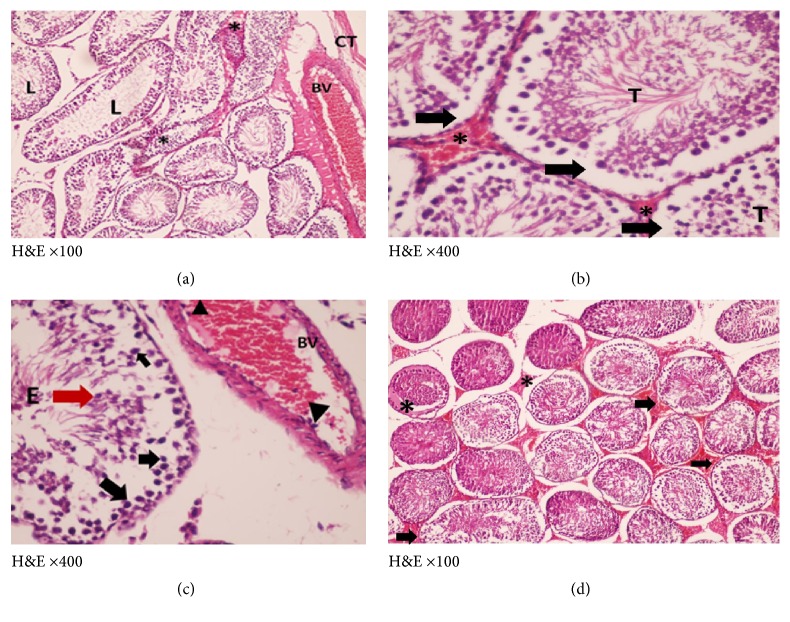
Photomicrographs of a cross section of adult rat tests of the I/R group showing (a) apparent congestion of the capsule (CT) containing dilated congested blood vessels (BV), some shrunken tubules (*∗*), and other tubules with wide lumina (L) (×100). (b) There is interstitial haemorrhage (↑) and excess acidophilic vacuolated exudates inside and outside the affected seminiferous tubules (*∗*) (×100). (c) Decrease in the primary spermatocytes (↑) and round spermatids (black rightwards arrow) series with degenerated cytoplasm and pyknotic nuclei (ghost cells). The lumen is full with fragmented degenerated exfoliated cells (E) and congested blood vessels (BV) with many dark stained kidney shaped nuclei (▲) of mononuclear inflammatory cell infiltrate (neutrophils) with margination from the lumen towards the blood vessels wall (×400). (d) Distorted seminiferous tubules (T), markedly disarranged spermatogenic cells, wide area of separation or detachment of the basal germ cells from the basement membrane (↑), and interstitial vacuolated acidophilic exudates (*∗*) ( ×400).

**Figure 8:**
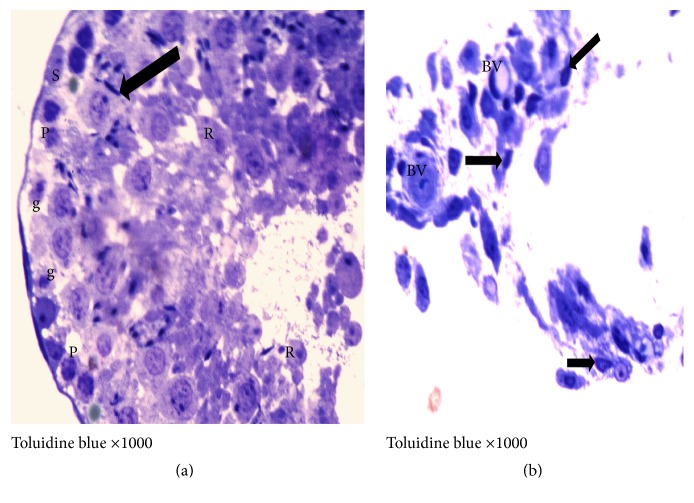
Photomicrographs of a semithin section of adult rat tests of I/R group showing (a) degenerated spermatogonia (g) with rarefied or vacuolated cytoplasm. There is incoherence of the degenerated primary spermatocytes (P) with pyknotic nuclei, loss of regular arrangement of round spermatids (R), and scanty elongated spermatids (↑) (×1000). (b) The majority of Leydig cells have pyknotic nuclei (↑) around the blood vessel (BV) (×1000).

**Figure 9:**
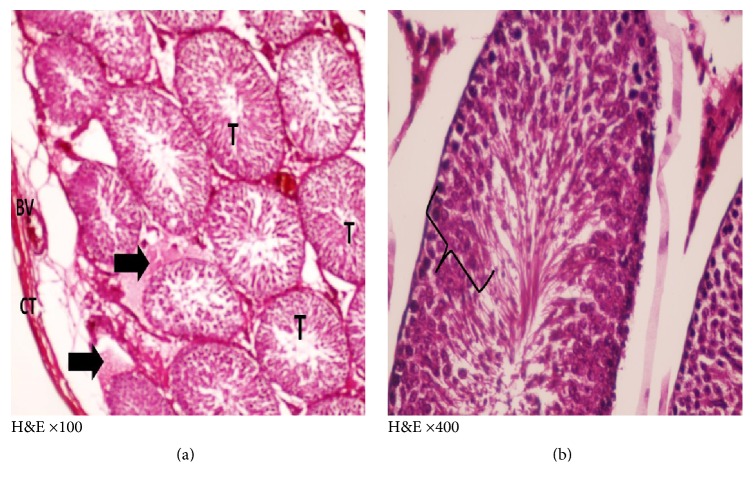
Photomicrographs of cross section of adult rat testis of the Ginkgo biloba treated I/R group showing (a) apparently decreased congestion of capsule (CT) and blood vessel (BV); the seminiferous tubules (T) display near normal configuration and arrangement. In some areas the interstitial space presents acidophilic vacuolated exudates (↑) (×100). (b) Seminiferous tubule with apparently normal thickness of germinal epithelium ( } ) and appearance. All spermatogenic stages are observed, but some vacuolation are still detected (×400).

**Figure 10:**
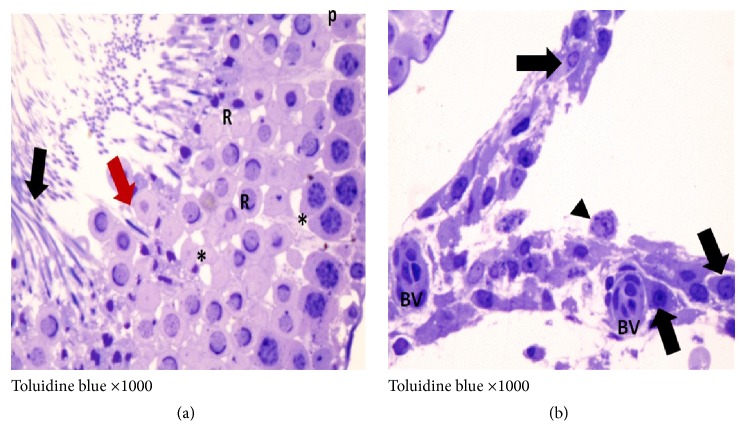
Photomicrographs of semithin section of the adult rat testis of Ginkgo biloba treated I/R group showing (a) restoration of all spermatogenic cells (black rightwards arrow) and sperms lining the luminal surface (↑). Few degenerated primary spermatocytes (P) are observed and round spermatids with characteristic acrosomal cap (R), with widened intercellular spaces between the regenerating germ cells (×1000). (b) Wide large interstitial space with 2 blood vessels (BV) surrounded by many apparently normal Leydig cells (↑) with macrophage cell with kidney shape nuclei in the space (▲) (×1000).

On the other hand, the I/R group showed moderate to severe degeneration and necrosis of the spermatogonia, as well as dead spermatids and interstitial edema. Within the tubules, desquamated epithelium, cellular debris, and fragments were, also, observed in addition to more prominent separation between germinative cells in the seminiferous tubules (Figures 7 and 8). In Gingko biloba treated I/R group, attenuation of the degenerative process of the testis to a mild degree of degeneration of the germinal epithelial lining of the seminiferous tubules was observed (Figures 9 and 10).

### 4.3. Immunohistochemical Results

**Figure 11:**
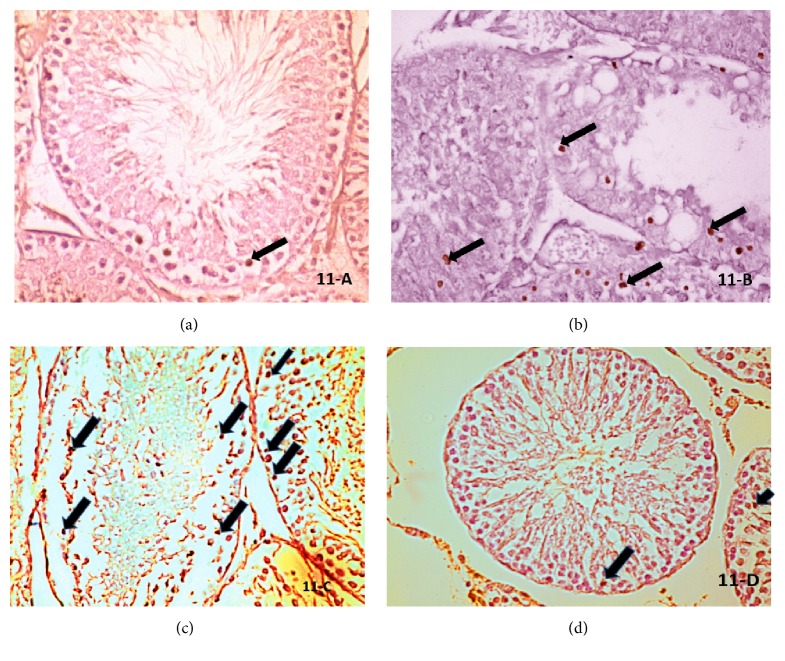
Photomicrographs of a transverse section in the adult albino rat testis showing (a) control group: normal spermatogenic cells with negative reaction. (b) Ischemia only group: multiple apoptotic germinal cells (↑) with positive Apaf-1 expression. (c) I/R group: many apoptotic germinal cells (↑) with positive reaction. (d) Ginkgo biloba treated I/R group**: **few apoptotic germinal cells (↑) with reaction (Apaf-1 stain ×1000).

Apaf-1 immunohistochemical stained sections of the control group revealed weak reaction of spermatogenic cells (Figure 11(a)), whereas there were multiple germinal cells with dark brown apoptotic nuclei in ischemia only group (Figure 11(b)) and I/R group (Figure 11(c)). Ginkgo biloba treated I/R group showed few scattered germinal cells with dark brown apoptotic nuclei (Figure 11(d)).

### 4.4. Sperm Abnormality Results

**Figure 12:**
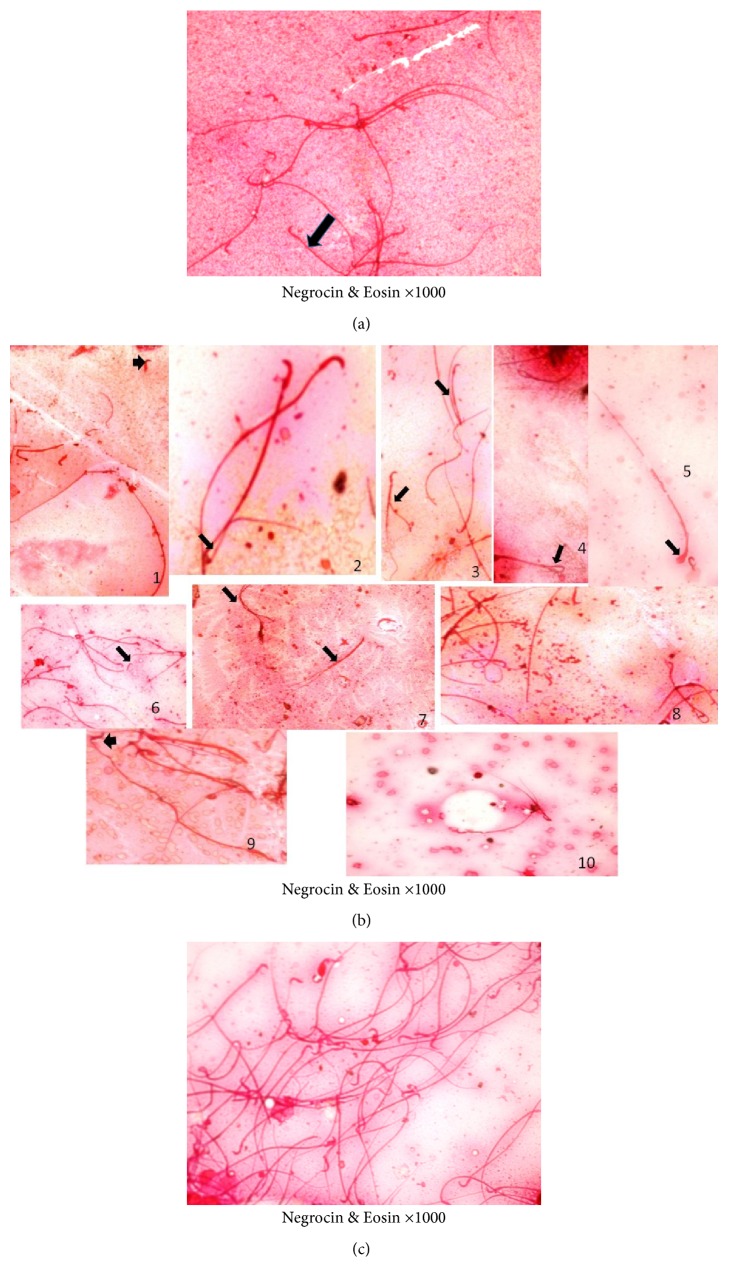
Photographs of sperms obtained from adult albino rat. (a) Control group showed normal mature sperms that appear with normal hook shaped heads and single long tail. (b) Some sperm abnormalities of ischemia only and I/R groups showed fragmented sperm, heads without tail or tails with absent heads (1), coiled tail (2), twisted or biffed tails (3), ballooned heads (4), spherical head (5), tails without heads (6), fusion of tails at different position (7), coiled tail (8), head with hook at wrong angle (9), or irregular heads(10). (c) Ginkgo biloba treated I/R showed mature sperms near normal sperms apart from few abnormal sperms.

The control group showed normal sperm morphology with prominent hook shape head and single long tail (Figure 12(a)); in ischemia only and I/R groups sperm abnormalities were markedly increased in the form of head anomalies (ballooned, spherical, heads without tails, hook at wrong angle, separated head fused with head of another sperm, and irregular heads) and tail abnormalities (coiled tails and fused tails at different position) (Figure 12(b)). Ginkgo biloba treated I/R group exhibited apparent reduction in the total sperm abnormalities compared to ischemia only and I/R groups (Figure 12(c)).

### 4.5. Morphometric Results

**Figure 13:**
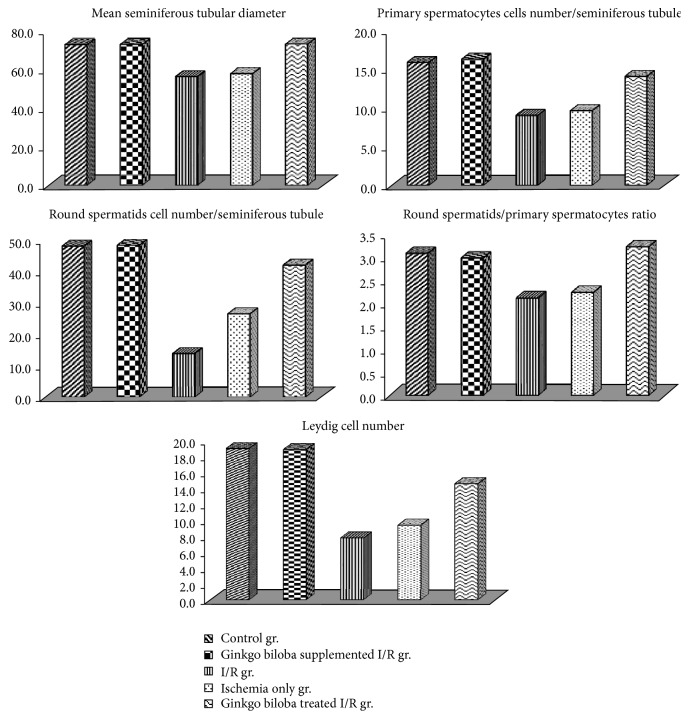
Changes in mean seminiferous tubular diameter primary spermatocytes cells number per cross section of a seminiferous tubule in the different studied groups, round spermatids cell number per cross section of a seminiferous tubule, and round spermatids/primary spermatocytes ratio and Leydig cells per space in the different studied groups.

Regarding the mean seminiferous tubular diameter (MSTD) for testes, as shown in Table 3 and Figure 13, the MSTD of both the I/R group and ischemia only group were significantly decreased (*P* < 0.001 for each) compared to the control group, while nonsignificant changes were observed in Ginkgo biloba supplemented and Ginkgo biloba treated I/R groups compared to control group. However, the MSTD was significantly increased in Ginkgo biloba treated I/R group compared to I/R group accompanied with insignificant change in ischemia only group when compared to I/R group, as shown in Table 3 and Figure 13.

Regarding parameters of spermatogenesis such as primary spermatocytes/HPF, the mean number of round spermatids/HPF, and the mean round spermatids/primary spermatocytes ratio/HPF, they were all decreased in both ischemia only and I/R groups compared to control group (<0.001 for each), while there were nonsignificant changes in Ginkgo biloba supplemented and Ginkgo biloba treated I/R groups compared to the control group. However, they were significantly elevated in Ginkgo biloba treated I/R group compared to I/R group (*P* < 0.002, <0.001, and <0.005, resp.). Although only the parameter of the mean of round spermatids number/HPF was significantly increased in ischemia only group compared to I/R group (*P* < 0.05), the remaining parameters of spermatogenesis were insignificantly changed in ischemia only group compared to I/R group, as shown in Table 3 and Figure 13.

As shown in Table 4 and Figure 13, there was a significant decrease in the mean number of Leydig cells/space in ischemia only group and I/R group when compared to the control group (*P* < 0.001 for each), whereas it was insignificantly changed in Ginkgo biloba supplemented and Ginkgo biloba treated I/R groups compared to the control group. However the mean number of Leydig cells/space was significantly increased in Ginkgo biloba treated I/R group compared to I/R group (*P* < 0.001).

## 5. Discussion

The ischemia/reperfusion was performed in this study by twisting the spermatic cord 720 degree, and a microvascular clamp was applied on it. Turner et al. [28] showed that 720-degree torsion induced ischemia sufficient to disrupt the seminiferous epithelium.

In the current study, the plasma-free testosterone level was reduced in ischemia only and I/R groups compared to control group. This is in accordance with Turner et al. [28], who found reduced testosterone level after repair of 1-hour, 720° torsion and was normalized days after the repair of torsion. However, Robert et al. [29] reported that testosterone did not show significant differences after 60 min of ischemia compared to basal values, and testosterone levels were decreased after 24 h of reperfusion. This discrepancy could be attributed to the suggestion of Azizollahi et al. [30], who reported that the changes in biochemical parameters occurred earlier in comparison to morphologic changes; thus determination of testicular damage following unilateral testicular torsion by biochemical analysis could be more informative than the histologic and morphologic studies alone in the acute phase of I/R models.

During the ischemic phase of I/R process, a hypoxic condition resulted from disrupted blood flow to testicular tissues leading to reduced tissue ATP production and increased calcium influx into the intracellular compartment elevating superoxide generator enzyme, in addition to chemotactic factors stimulation, thereby, migrating polymorphonuclear leukocytes to the ischemic region, which could generate superoxide radicals after reperfusion [31]. Despite the fact that blood flow restoration reversed the ischemic state, reperfusion could harm the tissue, and, particularly during the first 60–90 minutes, oxygen becomes abundant promoting the toxic burst of free oxygen radicals to invade neutrophils, macrophages, and residual parenchymal cells in the affected tissues [32]. Therefore, after testicular detorsion, tissue damage is aggravated by reperfusion injury [4]. Thus, in the current study, the periods of torsion and detorsion were 2 hours per each stage, because Turner and Brown [33] stated that one hour of minimum time caused testicular damage after experimental testicular torsion in the rat.

The decline in free testosterone level could be due to decreased Leydig cell count as observed in ischemia only and I/R groups, which is in accordance with Bergh et al. [34] and Creasy [35], who reported that I/R caused severe testicular damage, as well as disorganization in the form of multifocal germ cell degeneration and exfoliation. Turner et al. [28] reported that the minimal duration and degree of torsion might cause loss of rat spermatogenesis and could cause a significant reduction in testicular androgen production. The decline in testosterone level could be inversely related to the immediate torsion repair value after reperfusion suggesting that reperfusion injury could play a role in Leydig cell dysfunction, either acting directly, causing germ cell apoptosis or indirectly by inducing oxidative stress. However, the finding of Leydig cell damage disagrees with Shalaby and Afifi [36]. Fawcett [37] and Orsi et al. [38] reported that the seminiferous tubules exert a local regulatory effect on Leydig cells function through its suppression by inhibitory peptide produced by Sertoli cells. In the present study, Ginkgo biloba treated I/R group showed insignificant changes in free testosterone level compared to I/R group, together with preserved Leydig cell count compared to I/R group, which is similar to Kanter [15], who found that Ginkgo biloba increased testosterone level compared to I/R group and suggested that Ginkgo biloba could be protective against ischemia/reperfusion induced testicular damage by scavenging the free radicals.

The damaged Leydig cell together with decreased plasma level of free testosterone observed in the present study in I/R group could be attributed to apoptosis induced by I/R injury as evidenced by elevated IL1-*β*. Turner et al. [39] reported that the reperfusion induced injury to the testes causes significant increase in germ cell apoptosis due to high testicular oxidative stress following reperfusion.

In addition, FSH level was significantly elevated in I/R group compared to control group; however, it was insignificantly changed in ischemia only group compared to the negative control group. This rise could be attributed to the defective spermatogenesis in I/R group. In the present study, spermatogenesis was disrupted in ischemia only group and more affected in I/R group in the form of pyknosis and degeneration of the spermatogonia together with their separation from the basement membrane which were similar to Bergh et al. [34]. Genescà et al. [40] reported that reduced testicular blood flow could induce death among germ cells by apoptosis rather than by necrosis affecting primarily spermatogonia. Also, in the current study, many tubules were severely affected and were completely replaced by hyaline materials in ischemia only and I/R groups. Creasy [35] reported that the seminiferous tubules were avascular and metabolically active, depending on transport of oxygen and nutrients from the interstitial vasculature, putting them on the borderline of hypoxia and making them susceptible to blood flow reduction; thereby, the ischemic injury causes degeneration of germ cells. In the present study, the observed exfoliated cells in the lumen of the seminiferous tubules of both ischemia only and I/R groups agree with Creasy [35], who suggest a primary effect on the cell to cell junctions between Sertoli and germ cells.

In the testicular tissue, mean seminiferous tubule diameter (MSTD) is used to assess histopathological damage based on the evaluation of progressive degeneration of the germinal epithelium [41]. In the current study, a reduction in MSTD of the rat testes was observed in the ischemia only and I/R groups compared to the control group, which could be due to severe tubular atrophy, low spermatogenic epithelium, and disruption of spermatogenesis induced by ischemia and I/R [7]. However, it was preserved in the Ginkgo biloba treated I/R group compared to the control group, similar to Kanter [15], which could be due to the beneficial effects of Ginkgo biloba treatment on the spermatic cell preservation in the treated I/R group.

Also, the primary spermatocytes count, the number of rounded spermatids, and their ratio were decreased in ischemia only group and extensively in I/R group. Somwaru et al. [42] found that a high temperature induced a rapid increase in reactive oxygen in pachytene spermatocytes but not in round spermatids. Reyes et al. [43] mentioned that increased testicular temperature might cause the hypoxic changes in the testicles such as increased vascularisation, decreased testicular mass, and increased interstitial space. Therefore, the observed low level of plasma-free testosterone in the ischemia only and I/R groups may be the possible cause of spermatogenic impairment, similar to Singh et al. [44], who reported that testosterone was responsible for the activation and maintenance of spermatogenesis. In addition, O'Donnell et al. [45] found that testosterone could help in the maturation of round and elongated spermatids.

Congested testicular capsule was detected, in this study, in ischemia and I/R groups, which might be due to capillary dilatation and postcapillary venules slowing blood flow which became lodged with red blood corpuscles, as reported by Wheater et al. [46]. The present study showed haemorrhage between seminiferous tubules in both ischemia only and I/R groups while these changes were not observed in Gingko biloba treated I/R group, which is in accordance with Ünsal et al. [7], who attributed this to obstruction of venous backflow. Therefore, the protective effects of Gingko biloba could be due to its antioxidant property and increased tissue resistance particularly capillaries [7].

However, in the present study, there was no apparent affection of Sertoli cells. Turner and Miller [47] reported that the severe disruption of Sertoli cells was not a primary feature of testicular I/R injury. Lysiak et al. [48] suggested that Sertoli cells could survive after torsion repair and continue production of several proteins. This could explain the nonsignificant change of FSH in ischemia only group compared to the control group.

The disrupted spermatogenesis, in the present study, could be due to apoptosis and inflammation. As plasma levels of IL1-*β* and TNF-*α* as apoptotic factors and inflammatory mediators were significantly elevated in I/R group compared to the control group, however, they were significantly decreased in ischemia only group compared to I/R group. Similarly, Minutoli et al. [3] found that after a 24 h reperfusion, in I/R saline-treated rats, there was increased IL-6 and TNF-*α* expression and a marked damage in both testes. Thus, I/R could increase the proinflammatory cytokines TNF-*α* and IL-1*β*, suggesting a role for these cytokines as early mediators of injury in testis. TNF-*α* and/or IL-1*β* could recruit neutrophils in parenchymal testis veins [49], triggering germ cell apoptosis resulting in decreased germ cell mass and loss of fertility [50].

TNF-*α*is expressed as a transmembrane protein in pachytene spermatocytes, round spermatids, and testicular macrophages [51]. The release of TNF-*α*from the cell surface by shedding of the extracellular domain could be of value in the molecular I/R activated cascade. Also, the metalloproteases could have paracrine and autocrine signalling by controlling the ectodomain shedding of TNF-*α* [52], such as ADAM17, the main shedding part of TNF-*α*, expressed in germ cells, and its activity is necessary to induce apoptosis in physiological conditions [53]. Higher TNF-*α*level was found in semen samples from infertile patients compared to control subjects, suggesting its role in male subfertility specifically in I/R induced testicular changes [54]. Thus, testicular torsion could induce shedding of TNF-*α* as a primary response to hypoxia and oxidative stress [43].

Moreover, Ginkgo biloba treated I/R group showed significantly decreased plasma levels of IL1-*β* and TNF-*α* compared to I/R group and caused improved histological disruption of spermatogenesis observed in ischemia only and I/R groups. These findings agree with Serrano-García et al. [55], who reported that Ginkgo biloba administration reduced the ischemia and I/R induced damage in testicular tissue. Ginkgo biloba has antiapoptotic effects through the protection of mitochondrial membrane integrity, possibly by its flavonoid constituents [56], which was manifested in the present study, by decreased mitochondrial NAD^+^ in Ginkgo biloba treated I/R group. Also, Ginkgo biloba contains proanthocyanidins and ginkgolic acid which promotes vasodilation [56]; thus Ginkgo biloba was administered in this study 40 min prior to detorsion. Also, Guan et al. [57] found that the apoptotic index was decreased with the antioxidant and anti-inflammatory effects of the Ginkgo biloba treatment on the organs.

Erol et al. [58] evaluated germ cell apoptosis by using the Apaf-1 antibody. In the current study, immune-histochemical analysis of Apaf-1 protein expression revealed weak expression in testes of control group while it was strongly expressed in ischemia only and I/R groups with multiple apoptotic cells; however, mild expression was found in Ginkgo biloba treated I/R group. These findings agree with Lysiak et al. [59], who mentioned that loss of spermatogenesis and increased germ cell apoptosis might occur, even with successful surgical repair, due to increased testicular oxidative stress concomitant with reperfusion. A delayed upregulation of inflammatory mediators could cause tissue damage and organ dysfunction [60]. Yurtçu et al. [61] suggested that antioxidant agents could prevent testicular I/R injury. Hajra and Liu [24] showed that apoptosis led to increased Apaf-1 expression in the germ cells of rat testes. Yeh et al. [62] demonstrated that Ginkgo biloba protected against the oxidative and apoptotic actions of Doxorubicin on testes; thereby, Ginkgo biloba protected the rat testis against I/R injury evidenced in this study by less Apaf-1 expression in the treated I/R group.

Moreover, Altavilla et al. [63] reported that testicular I/R caused elevated production of reactive oxygen species (ROS) and induced growth factors including NF-*κ*B and IL1-*β*, which is responsible for the testicular atrophy, decreased blood flow, and impaired spermatogenesis as observed in this study. The preserved histological and less affected spermatogenesis found in Ginkgo biloba treated I/R group could be explained partially by significant decrease in IL1-*β* compared to I/R group. Similarly, Akgül et al. [64] found decreased apoptotic cells, eNOS, and iNOS in Ginkgo biloba treated I/R testes compared to I/R group. They reported, also, that Ginkgo biloba, as a free radical scavenger, could decrease apoptosis in testicular I/R injury.

In addition to I/R -induced apoptotic changes, the oxidative stress could be a contributing mechanism. Lysiak et al. [65] reported the adverse effects of oxidative stress on testicular function, including germ cell loss and disruption of the seminiferous epithelium, and that it might cause infertility [9]. Thus, inhibitors of oxidative stress could provide significant testicular salvage after torsion repair and organ reperfusion [66]. In the present study, there was diminished sperm production in ischemia only and I/R groups compared to the apparently normal production in both negative and positive controls. This finding is similar to Ramadan et al. [67]. Thus, the increased gonadotropins secretion, observed as increased plasma FSH level in I/R group, could be due to testicular damage caused by ischemia, causing diminished sperm production. In addition, Murugesan et al. [68] mentioned that mammalian spermatozoa, enriched with polyunsaturated fatty acids, could be susceptible to ROS attack. At the level of the isolated spermatozoon, ROS attack could induce lipid peroxidation and DNA fragmentation disrupting their motility [9]. Also, in the current study, abnormal sperm morphology was observed in ischemia only and I/R groups compared to the apparently normal sperms in both control and Ginkgo biloba treated I/R groups, which agrees with Acharya et al. [69] and Shalaby and Afifi [36], who stated that I/R increased the frequencies of sperm abnormalities. This might have resulted from the germ cells destruction after I/R injury and ROS release disrupting germ cells formation by producing morphologically abnormal sperms. Thus, the protective effects of Ginkgo biloba supplementation could be due to attenuation of lipid peroxidation process of the sperm cell membrane which was manifested by decreased testicular mitochondrial NAD^+^.

This study provides the novel finding of the role of mitochondrial NAD^+^ in generating a state of oxidative stress in experimental I/R in testis. Mitochondrial NAD^+^ was significantly elevated in I/R group compared to control group and to lesser extent in ischemia only group. Also, it was significantly decreased in ischemia only group compared to I/R group. This finding assures that I/R injury could cause more harmful effects of oxidative stress than that which occurs in ischemia only.

Therefore, oxidative stress could disrupt the steroidogenic capacity of Leydig cells [70] in addition to affecting the germinal epithelium differentiation into normal spermatozoa [71]. Agarwal et al. [72] demonstrated a correlative relationship between male infertility and oxidative stress markers in the ejaculate. The subfertility observed in the I/R group is similar to that in Anderson and Williamson [73] who reported that late presentation of or failure to diagnose and correctly manage testicular torsion resulted in subfertility.

The link between the oxidative stress and apoptosis was explained by Shiraishi et al. [74], who reported that germ cell necrosis could be an early stimulus in order to recruit neutrophils promoting oxidative stress and the induction of apoptosis. Although general caspase inhibitor and caspase-9 inhibitor prevent germ cell apoptosis in I/R testis, the necrosis could, also, contribute to germ cell derangement. I/R could cause the neutrophils recruitment to subtunical venules in the testis. In the present study, there was mononuclear inflammatory cell infiltrate in the interstitial space and it marginated from the blood vessels lumen towards the wall in ischemia only and I/R groups. Lysiak et al. [59] reported that I/R injury could cause endothelial cells damage following I/R resulting in the cell surface expression of cell adhesion molecules involved in neutrophil recruitment causing increased adhesion to the testicular venous endothelium.

Kroemer et al. [75] mentioned that the early phase of both cell death modes could include changes in mitochondrial membrane permeabilization (MMP) which is caused by opening of a nonspecific pore in the inner mitochondrial membrane, known as the mitochondrial transition pore (MTP), thereby allowing the passage of any molecule of >1500 Da across the inner mitochondrial membrane, causing rapid protons passage associated with mitochondrial depolarization and uncoupling of oxidative phosphorylation. In addition, the equilibration of small solutes across the inner mitochondrial membrane caused high concentrations of proteins in the matrix exerting a colloidal osmotic pressure responsible for the extensive mitochondrial swelling associated with MTP opening [76]. The depleted ATP levels could be totally present in prolonged opening of mitochondrial membrane resulting in cell necrosis. However, when it transiently opened, there could be activation of intrinsic pathway or mitochondrial-mediated apoptosis through the apoptogenic protein release such as cytochrome C [77]. Thus, the finding of increased mitochondrial NAD^+^ in I/R group could explain the disrupted spermatogenesis observed in this group. Reyes et al. [43] reported that spermatogenesis, an extremely active replicative process, consumes high rates of mitochondrial oxygen consumption by the germinal epithelium. The testicular oxygen tension could be low and could be reduced more in blood flow decline to the testis such as in varicocele or testicular torsion. Sheweita et al. [78] mentioned that the ROS production by a sperm is a normal physiologic process and ROS was produced by a variety of semen components, including immotile or morphologically abnormal spermatozoa, leukocytes, and morphologically normal but functionally abnormal spermatozoa.

Also, the finding of increased mitochondrial NAD^+^ in I/R group could explain the observed decrease in free testosterone in I/R group. Turner et al. [28] reported that I/R injury could cause a loss of testicular testosterone secretion, which might be temporary and defective spermatogenesis induced by intratesticular ROS [47], stimulating germ cell-specific apoptosis [79], which is not reported in Sertoli cells or Leydig cells after testicular torsion repair, although ROS disrupted Leydig cell steroidogenesis through perturbation of the mitochondrial membrane [80]. This could be the reason for the temporary decline in steroidogenesis that occurred after torsion repair in the rat [28], explaining the elevated mitochondrial NAD^+^ and decreased free testosterone level in plasma observed in the present study.

While mitochondrial NAD^+^ level was significantly reduced in Gingko biloba treated I/R group compared to the I/R group. Akgül et al. [81] found that Ginkgo biloba decreased I/R induced testicular injury examined by malondialdehyde, nitrate, and nitrite levels. This novel finding of mechanistic tool of Gingko biloba in testicular I/R could enrich the antioxidant property of Gingko biloba.

In conclusion, this study demonstrates that ischemia/reperfusion adversely damages testicular tissue and significantly reduces sperm production through inducing oxidative stress and apoptosis, while Ginkgo biloba treatment effectively attenuated these changes. Therefore, the aforementioned decline in plasma-free testosterone level together with increased plasma FSH level caused by elevated mitochondrial NAD^+^ and apoptotic factors, IL1-*β* and TNF-*α*, could result in the state of subfertility induced by ischemia/reperfusion. In addition, the mitochondrial NAD^+^ could be a mechanism of Ginkgo biloba in ameliorating I/R induced subfertility.

## Figures and Tables

**Table 1:** Hormonal changes in the different studied groups.

	Control group (10)	Ginkgo biloba supplemented group (10)	I/R group (10)	Ischemia only group (10)	Ginkgo biloba treated I/R group (10)
Plasma-free testosterone level (nanogm/ml)	142.5 ± 29.83	127.5 ± 18.88	30 ± 11.55	52.5 ± 10.31	113.33 ± 34.8
*P*		*NS*	*<0.005*	*<0.01*	*NS*
*P* ^*∗*^				*NS*	*<0.05*

Plasma FSH level (mIU/ml)	1.76 ± 0.03	1.86 ± 0.03	2.12 ± 0.17	1.9 ± 0.07	1.92 ± 0.05
*P*		*NS*	*<0.02*	*NS*	*NS*
*P* ^*∗*^				*NS*	*NS*

*P*: significance by LSD at *P* < 0.05 from control group.

*P* ^*∗*^: significance by LSD at *P* < 0.05 from I/R group.

NS: not significant.

**Table 2:** Changes in oxidative stress marker mitochondrial NAD^+^ and inflammatory and apoptotic markers in the different studied groups.

	Control group (10)	Ginkgo biloba supplemented group (10)	I/R group (10)	Ischemia only group (10)	Ginkgo biloba treated I/R group (10)
Mitochondrial NAD^+^ (nmol/mg tissue protein)	6.5 ± 1.09	7.81 ± 1.57	16.83 ± 1.53	11.29 ± 0.92	8.67 ± 1.58
*P*		NS	<0.001	<0.05	NS
*P* ^*∗*^				<0.01	<0.001

Plasma TNF-*α* (ng/ml)	35.15 ± 0.51	35.71 ± 0.40	107.65 ± 19.78	36.38 ± 1.4	55.88 ± 6.22
*P*		*NS*	*<0.001*	*NS*	*NS*
*P* ^*∗*^				*<0.001*	*<0.001*

Plasma IL-1*β* (pg/ml)	59.39 ± 0.53	55.26 ± 1.79	97.71 ± 19.08	60.57 ± 1.17	47.04 ± 0.07
*P*		NS	<0.01	NS	NS
*P* ^*∗*^				<0.02	<0.002

*P*: significance by LSD at *P* < 0.05 from control group.

*P* ^*∗*^: significance by LSD at *P* < 0.05 from I/R group.

NS: not significant.

**Table 3:** Changes in the mean diameter of the seminiferous tubules (micrometers), primary spermatocytes number/high power field (HPF), round spermatids number/HPF, and round spermatids/primary spermatocytes ratio/HPF in the different studied groups.

	Control gr.	Ginkgo biloba supplemented group	I/R gr.	Ischemia only gr.	Ginkgo biloba treated I/R gr.
Mean seminiferous tubular diameter	72.99 ± 4	73.12 ± 3.9	56.43 ± 2.16	58.04 ± 3.1	73.31 ± 1.38
*P*		NS	<0.001	<0.001	NS
*P* ^*∗*^				NS	<0.001

Count of primary spermatocytes	15.8 ± 1.09	16.2 ± 1.2	9 ± 1.2	9.6 ± 1.11	14 ± 2.1
*P*		NS	<0.001	<0.001	NS
*P* ^*∗*^				NS	<0.002

Number of round spermatids	47.8 ± 1.83	48.1 ± .06	13.8 ± 4.12	26.4 ± 3.45	41.8 ± 1.72
*P*		NS	<0.001	<0.001	<0.001
*P* ^*∗*^				<0.05	<0.001

Round spermatids/primary spermatocytes ratio	3.06 ± 0.02	2.96 ± 0.05	2.1 ± 0.02	2.22 ± 0.02	3.2 ± 0.03
*P*		NS	<0.001	<0.001	NS
*P* ^*∗*^				NS	<0.005

*P*: significance by LSD at *P* < 0.05 from control group.

*P* ^*∗*^: significance by LSD at *P* < 0.05 from I/R group.

**Table 4:** Changes in the means of Leydig cells number/high power field (HPF) in all studied groups.

Leydig cells	Control gr.	Ginkgo biloba supplemented group	I/R gr.	Ischemia only gr.	Ginkgo biloba treated I/R group
Mean ± SEM	19 ± 2.25	18.87 ± 3.16	7.8 ± 0.09	9.4 ± 1.28	14.6 ± 1.21

*P*		NS	<0.001	<0.001	NS
*P* ^*∗*^				NS	<0.001

*P*: significance by LSD at *P* < 0.05 from control group.

*P* ^*∗*^: significance by LSD at *P* < 0.05 from I/R group.

## Abbreviations

Apaf-1: Apoptosis protease-activating factor 1
I/R: Ischemia/perfusion
MMP: Mitochondrial membrane permeabilization
MSTD: Mean seminiferous tubule diameter
MTP: Mitochondrial transition pore
NO: Nitric oxide
ROS: Reactive oxygen species.
